# Re-engaging with arts and cultural activities at the Life Rooms: ‘It’s given me spring’

**DOI:** 10.1186/s12906-024-04539-6

**Published:** 2024-06-15

**Authors:** Joanne Worsley, Josie Billington, Ekaterina Balabanova

**Affiliations:** 1 Department of Primary Care and Mental Health, University of Liverpool, Liverpool, UK; 2 Department of English, University of Liverpool, Liverpool, UK; 3 Department of Communication and Media, University of Liverpool, Liverpool, UK

**Keywords:** Renewed access, Arts engagement, Reconnecting, Online provision, Public mental health, Wellbeing

## Abstract

**Background:**

Mersey Care NHS Foundation Trust’s Life Rooms initiative is an established and successful model of integrating arts and culture within NHS provision. In the face of COVID-19, the Life Rooms was restructured to provide a full suite of online resources. Following the lifting of restrictions, in-person arts provision returned to the Life Rooms sites. Additional evidence in respect of the impact on mental health and wellbeing of the return to in-person arts and cultural activities provided by the Life Rooms, as well as the relative merits of online and in-person arts and cultural activities, is needed to inform future planning around in-person, online, and/or hybrid provision.

**Methods:**

Interviews with practitioners delivering cultural and creative courses at the Life Rooms (*n* = 8) and users of the Life Rooms (*n* = 5) were conducted to explore the impact of the return to in-person arts provision at the Life Rooms, as well as the merits of online and in-person arts provision. Data were analysed using thematic analysis.

**Results:**

Three overarching themes were identified: ‘Provision, access, and reach’; ‘Value of arts and creativity’; and ‘Challenges with the Life Rooms model in the new normal’. The findings demonstrate the critical role of arts and cultural provision in providing stigma-free environments to reconnect the vulnerable and isolated. As re-engagement remained slow, there is a need to be responsive to hesitation around re-engaging in-person. The Life Rooms online learning offer remained vital for those who are vulnerable or otherwise unable to access in-person activities.

**Conclusions:**

As our findings show a demand for maintaining online provision that enables accessibility together with in-person provision that boosts community connectedness, ensuring continued online access alongside in-person delivery should be prioritised. As mental health demands could continue to grow in coming years as the pandemic evolves, in-person arts engagement could have an important role in meeting mental health needs.

**Supplementary Information:**

The online version contains supplementary material available at 10.1186/s12906-024-04539-6.

## Background

The COVID-19 pandemic has had a profound effect on all aspects of society, including mental health [1]. Recent research on the impact of COVID-19 has highlighted the increased mental health risks amongst the general UK population [2]. The Centre for Mental Health estimates an extra 10 million people are in need of mental health support in England as a direct consequence of the pandemic [3]. The longer-term consequences on wellbeing of COVID-19 for vulnerable groups, including those with pre-existing mental health conditions, are concerning [1].

There is a substantial and growing body of evidence suggesting that the arts can support mental health and wellbeing [4]. Engagement with the arts can have preventative benefits, such as enhancing wellbeing, reducing the impact of trauma, and decreasing the risk of cognitive decline in older adults [5]. Research has also demonstrated protective associations between engaging in participatory arts activities and the management or treatment of mental health difficulties, such as anxiety, depression, and schizophrenia (e.g., [6–8]). Participatory arts activities are beneficial for mental health and wellbeing as these activities are often undertaken in community-based groups. In a recent study, participation in local community arts groups (e.g., choirs, dance, photography, theatre, music groups) was associated with higher levels of life satisfaction, positive affect, purpose in life, and perceived mastery [9]. According to a recent systematic review, regular participation in community-based music and singing interventions enhances and maintains subjective wellbeing for older adults [10]. When engaging in arts-based activities, including dance, music, and visual arts, older adults experienced more positive affect, personal growth, and increased meaningful social interactions [11]. As such, the arts have been conceptualised as multi-modal health activities, providing a vehicle for people to engage in multiple different health promoting activities, including social integration, physical activity, and cognitive stimulation [5].

With some of the poorest mental health outcomes in the country [12], and one of the richest concentrations of culture in the UK, the Liverpool City Region (LCR) has a pioneering history of harnessing arts for mental health care through partnerships between arts and health providers [13–16]. Mersey Care NHS Foundation Trust, a provider of specialist and community mental health services across North West England, has a history of working in partnership with the Voluntary, Community, Faith and Social Enterprise (VCFSE) sector to enhance social inclusion and enable people to optimise their health by enriching their life experiences in the broadest possible sense [13–16]. Since 2008 (Liverpool’s European Capital of Culture year), Mersey Care NHS Foundation Trust has fostered community, creative, and cultural partnerships that are now integral elements of the care offer.

The Life Rooms community-centred service was introduced by Mersey Care in 2016 to support the health and wellbeing of service users, carers, and local communities. Based on a social and preventative non-clinical approach, the Life Rooms integrates public, private, and the VCFSE sector to improve population health [16, 17]. The Life Rooms Walton, Southport, and Bootle provide services to individuals residing in both Sefton and Liverpool metropolitan boroughs. Individuals who access the Life Rooms include Mersey Care service users and carers, people referred from primary care or public or third sector organisations, and the general public. As some individuals who access the service may be vulnerable due to a wide range of social, emotional, behavioural, and cognitive reasons, the service operates in the recovery arena whilst also working in a preventative way to empower people to live happier and healthier lives and potentially mitigate the need to access formal services. Although Life Rooms staff members, referred to as learning facilitators, provide ‘in-house’ learning opportunities ranging from topics such as understanding and managing mental health difficulties through to social and creative offerings, the service also has over 100 partnerships with statutory, private, and voluntary sector organisations. Working in partnership with a number of creative and cultural providers, the Life Rooms is an established and successful model of integrating arts and culture within NHS provision [16]. Examples of creative and cultural providers working in partnership with the Life Rooms include Liverpool Philharmonic (a concert hall), Liverpool Everyman and Playhouse (a theatre), The Reader (a shared reading organisation), and Movema (a grass roots dance organisation). In the face of COVID-19, in-person arts provision at the Life Rooms was ceased. The Life Rooms launched an online learning platform in October 2020, with creative and cultural courses being delivered exclusively online by creative practitioners (i.e., those who exhibit and use creative practices in their professions). Following the easing of restrictions, in-person arts provision re-commenced at the Life Rooms during Winter 2021.

The present study explored the impact, from the service user and practitioner perspective, of the return to in-person arts provision at the Life Rooms, as well as the merits of online and in-person arts-in-health provision. We focused on people’s experiences of returning to in-person arts-in-health provision at the Life Rooms, as well as of accessing continued hybrid and online arts and cultural activities. The purpose of this study was to inform future planning around in-person, online, and/or hybrid provision.

## Methods

This study is part of a larger longitudinal project examining the impact on arts providers, practitioners and beneficiaries of restricted access to arts and cultural activity, and the impact of renewed access^1^.

### Ethical approval

The project was deemed a service evaluation according to the local NHS Trust Research & Development department and the Health Research Authority decision tool. Approval for the project was obtained from the NHS Trust Research & Development department (Ref: SE2022-05). Ethical approval was received from the Central University Research Ethics Committee (7994). All methods were carried out in accordance with relevant university guidelines and regulations.

### Participants

Thirteen participants, including practitioners delivering cultural and creative courses at the Life Rooms and users of the Life Rooms, participated. Reflections from eight creative practitioners (six females, two males) and five users of the Life Rooms (three females, two males) were gathered using semi-structured interviews. Creative practitioners delivering courses at the Life Rooms were invited to participate via email. Users of the Life Rooms were invited to participate via an email, sent by the Life Rooms on behalf of the research team, which provided a brief overview of the study as well as the first author’s contact details. Individuals who were interested in taking part contacted the first author via email. All individuals who responded to the email were invited to participate and were sent a copy of the participant information sheet. All participants provided written informed consent.

### Data collection

Semi-structured telephone or video call interviews using a prepared topic guide were conducted between March and April 2022. Interviews with users of the Life Rooms explored their experiences of participating in cultural and creative courses at the Life Rooms and via the Life Rooms online learning platform (see Supplementary File 1). Interviews with practitioners explored their experiences of delivering creative courses, with a focus on the return to in-person provision (see Supplementary File 2). Interview duration ranged from 30 min to one hour. All interviews were conducted by the first author (trained and experienced in qualitative data collection and analysis). Interviews were audio recorded and transcribed verbatim.

### Data analysis

Data were analysed using the thematic analysis procedure outlined by Braun and Clarke [18]. Thematic analysis is a qualitative method that aims to identify, analyse, and report recurrent themes in data [18]. Although there were some pre-determined areas the researcher wanted to explore, a largely inductive approach was used to reflect on unexpected concepts within the data. Line-by-line coding, undertaken by the first author, ensured that data were not overlooked. The wider research team met frequently throughout this process to discuss resultant coding and examine developing impressions of the data. The resultant codes were arranged into wider themes and subthemes by the first author. Initial themes captured by coding were refined during discussions with the wider research team to produce the final themes and subthemes. This process ensured that the final themes and subthemes were not just the personal interpretation of one team member.

## Results

Our analysis offers three broad themes, titled: ‘Provision, access, and reach’; ‘Value of arts and creativity’; and ‘Challenges with the Life Rooms model in the new normal’ (see Table 1). The creative practitioners’ quotes are denoted by ‘CP’ and the service users by ‘SU’.

**Table 1:** Overarching themes and subthemes

Themes	Subthemes
Provision, access, and reach	Renewed accessibility
	Continued need for online provision: ‘*I might be very distressed if it disappeared*’
Value of arts and creativity	Promotion of psychological wellbeing through artistic and creative pursuits
	Role of creativity and the arts beyond the pandemic: ‘*It’s all about connecting people*’
Challenges with the Life Rooms model in the new normal	Insufficient resources
	Marketing and promotion
	Inadequate feedback mechanisms

## Provision, access, and reach

### Renewed accessibility

In the face of the COVID-19 pandemic, the Life Rooms was restructured to provide a full suite of online resources as traditional modes of provision were ceased. As people were deprived of in-person engagement opportunities, participants described the joy and celebration which accompanied the return to the Life Rooms:

*When it reopened and I went back for the first time, I felt quite giddy and joyous and happy that this space was open for people in the community again* (SU3).

*It’s just like spring. It’s not spring season, but spring in my life* (SU5).

Creative practitioners were acutely aware of the value of returning to in-person arts provision, with many highlighting ‘getting together’ in a physical space as a key ingredient for wellbeing, as ‘*need[ed] for our mental health*’ (CP4). Indeed, online provision for certain arts activities may not provide as ‘strong’ wellbeing benefits as in-person engagement, as online sessions are less personal and there are fewer social benefits:

*The human connection of being in a room with other people is massive, especially for this art form of drama. It’s massively about the senses. You see people, you’re next to people, you’re in the same room. It’s not vicarious connection through a screen, it’s person-to-person connection in a room. The impact on wellbeing is stronger in the room. In terms of my exercises, I can do more things in-person, because that’s how drama naturally is… Being in-person you connect physically. You’re actually interacting with people in the flesh, and that really picks people up a lot*… *Those who did come were amazed and delighted… There was a joy around being able to do these [drama] exercises in-person. I think it was very fulfilling after such a long time of isolation and separation. I think there was a lot of fulfilment and satisfaction and laughter* (CP3).

Some service users also expressed a clear preference for in-person arts provision, not only for the social benefits, but as offering a meaningful reason to leave their house, which may inspire confidence. Resuming in-person activities at the Life Rooms provides an opportunity to meet new people and forge new connections with similar others, whilst also enjoying arts and culture in a shared physical space. Participants attributed the return to the Life Rooms a rediscovery of motivation and a means to overcome loneliness:

*I got fed up with Zoom. I didn’t feel the connection. Particularly I’d say with arty or creative activities. In the main it is something where you’re connecting with people, your eye contact, you’re giving your story. Storytelling, for example, on Zoom, I just don’t think that works at all*… *I think it [referring to in-person arts provision] gets you out the house. It makes you do something, whether it’s walking to or getting the bus. And that’s one of the big things for me that I’m enjoying now, and really, really getting excited about getting back out again. It’s getting me out of bed. It’s a different thing to be going out for a purpose* (SU3).

*It was actually making me get out there and do it, you know, go into a room full of people I didn’t know apart from one, getting your confidence back about going out again* (SU5).

*I would prefer in-person. I’d rather spend the money and travel for two hours and be there in-person rather than sit in my house isolated* (SU4).

In addition to working with others in the same room, service users also appreciated being able to utilise the Life Rooms spaces again, finding value in the surrounding social opportunities available. In fact, for some service users, socialising with others face-to-face was given precedence over engaging in the arts activity, highlighting the importance of the collective and social aspects of in-person engagement:

*One of the virtues of meeting in-person is not only the physicality of working with people that are there in the room with you, but there’s also the peripheral chit chatting and having a cup of coffee and that sort of element, which you cannot do on Zoom… I can well understand somebody who is lonely or lives by themselves would most welcome face-to-face sessions, because then you have all that peripheral interaction* (SU2).

*I have just lately gone back a couple of times, and you sit there, and I will start chatting to someone or they will start chatting to me. I think that’s a very therapeutic environment… So, you might have a chat before and a little chat afterwards, and you might connect with individuals. I don’t think that happens on Zoom* (SU3).

The Life Rooms spaces therefore enrich provision in a way that was lost when connecting remotely, with one practitioner acknowledging that the ‘*community and communal feeling of togetherness continues before and after the session*’ (CP3) when in-person. In-person engagement opportunities are therefore an important catalyst for ‘*a real sense of community*’ (CP2):

*In Southport [Life Rooms]it was really uplifting for them to be able to share moments with each other and just to be able to come back together and nurture that sense of community that goes with the Life Rooms*… *It was interesting to see that when I arrived around quarter to 10, most of them were already all there sharing a bun and a cup of coffee and waiting to start* (CP2).

*If you’re in person, you come out of a session in Walton [Life Rooms], then you go into the main lobby area, which is a beautiful space, and you have a cup of tea, you carry on chatting, I see people chatting with a cup of tea before the session… But online you’re still isolated. You switch your computer on, you connect but then you switch your computer off, and you’re isolated again. So, definitely, I think people get a lot more out of in-person just because of the other effects of being with other people, before and after*. *Participants who go to the Life Rooms and basically hang out there all day, they’ve got access to hot drinks, other people, books, other sessions, members of staff, professional development, as well as mental health intervention sessions… It’s a community centre the Life Rooms… You see people saying hi to other people, there’s a sense they know each other, and they’re glad to see each other, and they’re glad to be in the same space and the same company as other people* (CP3).

As the Life Rooms caters for vulnerable populations, some of whom may be susceptible to severe COVID-19 symptoms and hospitalisation, safety measures, such as social distancing and limited class numbers, remained in place (‘*The windows are always open, and the doors are open and it’s quite a large room* (CP1)). Such measures helped to instil a feeling of safety:

*Everything there was perfect. There was sanitisation. The course wasn’t over full. It was in an open space, so you’ve got plenty of ventilation. There was nothing to worry about. Nobody was worried. Nobody wore masks. [Name of facilitator] wore a mask but that was her prerogative. But one or two people had disabilities and they weren’t stressed out at all. So, from a safety point of view, everything was adhered to. It was just perfect* (SU5).

Despite safety measures remaining in place, the return to in-person provision at the Life Rooms has been cautious, with practitioners describing the transition as gradual:

*People who have just come back from doing the online, coming back into in-person, it takes them time to get used to come out of the mask. It’s a personal choice… Everyone has to ease themselves through the transition* (CP4).

*It’s a slow process. I think there’s a lot of anxiety about organising to come to things. People got so used to their life being restricted* (CP1).

Although Life Rooms staff members have reached out to those who accessed the service before the pandemic, the number of people accessing the service remains lower than before the crisis:

*Because of the restrictions of COVID, the room that we’re in can have 12 people, but we’ve never had that. The most I’ve had since I’ve been back is about six. Now prior to [the pandemic], I would have about 13 or 14 in the shared reading group… Generally, the people coming into the Life Rooms, footfall, it’s nothing like it was… Life Rooms have tried to reach the people who used to come. I feel quite sorry and sad about it because it was just brilliant* (CP1).

*I’d say on average there are about five people coming… Numbers are still relatively small, and I’m not quite sure why that is. I think it’s a combination of some people have personal boundaries that they didn’t feel that they wanted to go back in-person [and] the Life Rooms publicity… I don’t think the word got out that in-person was happening again. I think there was a lot of hesitation. So, numbers were small* (CP3).

### Continued need for online provision: ‘I might be very distressed if it disappeared’

Re-engaging with arts and cultural activities at the Life Rooms involves risk assessment, especially for those who are susceptible to experiencing severe COVID-19 symptoms and hospitalisation. Despite the vaccination programme, a sense of caution and anxiety remained around attending in-person courses at the Life Rooms:

*Since the Life Rooms reopened, our attendance has still been on Zoom… because both of us are immunosuppressed. So that would mean that really our attending at the moment when COVID numbers are so high would be extremely risky* (SU1).

*The convenience of a Zoom session, combined with the fact that at the moment, we are still cautious of mixing with people means that it’s good for us* (SU2).

The continuation of online provision has therefore been vital as a means to reach people who are vulnerable or otherwise unable to access in-person activities. Such provision remains important for those with underlying health conditions or mobility issues, those who cannot afford travel costs, and those who experience social anxiety. In addition, online access has made it possible for first-time beneficiaries to experience creative and cultural courses as part of the Life Rooms offer:

*I think now, particularly with us both being immunosuppressed and ageing and potential new surgeries coming along, I will still wish to maintain Zoom if that’s possible. I might be very distressed if it disappeared* (SU1).

*If you have access to internet, broadband and technology, it can engage people who can’t physically move. So, it’s a very democratising process, it doesn’t cost you money to get on the bus, you don’t have to wrap up against the weather, you can literally be in your room… People can switch their computer on, and engage with other human beings in an art form. Online is really important for reaching those people who can’t easily get into a centre [referring to Life Rooms]* (CP3).

The importance of maintaining online provision to ensure inclusivity was recognised in relation to a range of issues, including both health and economic factors, which makes re-engaging with in-person arts provision at the Life Rooms challenging for certain groups:

*Even though we have the vaccinations, they have a waning and lesser effect as you’re ageing, but you still want to have access to the creative aspects of what the Life Rooms can offer as well as the socialisation. So you really have to have both [in-person and online], you have to have the Life Rooms open as a hub, where people can go, but equally for people who are unwell or immunosuppressed and actually have COVID, but are still able to join in, carrying on having at least some sessions or every other session, potentially a mixed session where it can be both Zoom and in-person… The Life Rooms is something that we have benefited from hugely and I would hate to think that our access would be lost in the future* (SU1).

*There are still people who really struggle to be in a room with people, there are so many… We need to be sensitive enough to understand that there are a group of people that really still want to have this [referring to online provision], and they are still doing baby steps to get out, so we need to provide that to them and help them to make that transition from the online to the outside because everyone is different* (CP4).

As well as catering for those neither able nor ready to return to in-person sessions *(‘I’m very conscious that there were a number of people who were regulars at the online group who are shielding*^2^*’ (*CP8)*)*, online provision was perceived as an enabler to people engaging in in-person provision. Attending online sessions provides an opportunity – or ‘gateway’ (CP8) - for service users to familiarise themselves with the course and facilitator before transferring to in-person provision:

*If someone was apprehensive about coming back into the centres [referring to Life Rooms], - or they have got some anxiety because of COVID and I’m sure there is mild post-traumatic stress, particularly in the older generations - and they did an online course, they could then be offered a hybrid course to slowly integrate into coming back into the class and then go to fully in the class… So, six weeks online, six weeks hybrid, six weeks in class* (SU4).

*If you’ve got people that are maybe fearful of travel and isolated and vulnerable, of which there are lots of people, it’s [referring to online provision] a really good way of connecting with people that aren’t ready to leave the house and in terms of mental health, that is on the rise… For me, it would be a way of building relationships with a view to help, to aid the transition to being in a physical space. Eventually, you could do some really great stuff in-person… I know from working with people with mental health in different settings that everybody says, once they’ve done it, they feel better, but not everybody has the motivation to do it. And the journey from getting from home to the centre [Life Rooms] seems like such a big step. But if there was support on Zoom, you could see if that does address the isolation* (CP7).

Nevertheless, initial enthusiasm for online provision waned (‘*The numbers of attendees, has gone down over the last year or so. From regularly being anything up to 10… it’s now down to regularly three or four’* (SU2)), and as many arts and cultural courses have re-commenced in-person, opportunities to participate in online courses have reduced:

*Through the lockdown, I taught Life Rooms dance. I did sessions about meditation… I loved those classes because people started to open up and share how they felt and there was a massive shift in them… Unfortunately, the mindfulness class, never continued. It’s a shame because when I finished the class, the people were like are you going to continue and then it never continued. We were just directed into the [in-person] dance classes* (CP4).

*There was a point where there were three exercise classes per week on mindful stretching or just a general fitness one. For us, it was still a mobility and a toning exercise class, sometimes using weights. All the classes tailed off significantly. So much so for the last couple, it was just me. So, they’ve now stopped entirely as far as I can see. So, they only take place now within a Life Rooms location* (SU2).

The issue of financial viability was acknowledged, as there are costs associated with providing online provision, even when these sessions are not well attended:

*It’s a difficult situation because anytime you provide an arts provision, you have to pay for the practitioner and practitioners of a certain calibre have a certain wage. So, it is going to be expensive. There is going to be budgetary constraints. Like, is it worth paying someone X amount per hour to provide an online session for two people… Even if no one turns up, you still have to pay that practitioner, it’s high risk at the moment, because not many people are coming on to online stuff* (CP3).

In light of these concerns, it was suggested that the Life Rooms could consult service users via an anonymous survey to identify and accommodate their preferences:

*It’s about asking them what is their preference at the moment. Do you feel like you are ready to go out? Some will say ‘yes I’m fine’ and others will say ‘I would prefer to do it at home’. Then maybe you will get a group of people doing it from home and a group of people doing it out. I know it’s probably easier said than done but if you can address both, you should try to be flexible and accommodate all. You are trying to reach out and help people. It’s not about the person coming in to teach, it’s about them [‘the people’] because that’s what Life Rooms is, you’re trying to help people and cater for them, if you’re not flexible in providing that then you won’t have as many people as you would wish for* (CP4).

*I definitely feel like asking those questions [referring to engagement preferences] to the people that are using the space, because it’s all well and good me saying I prefer a dance practice that’s in a physical space… So, it would be about listening to the people that you are working with, but it would also be listening to perhaps people that were engaged with the service before and then not engaged now* (CP7).

## Value of arts and creativity

### Promotion of wellbeing through artistic and creative pursuits

Users of the Life Rooms recognised the physical health, mental health, and wellbeing benefits of engaging in arts provision, and pursued creative and cultural courses for these reasons. Participants reported that both forms of arts provision (online and in-person) helped them to maintain positive mental health and wellbeing. In particular, creative courses helped people use certain psychological strategies for coping and emotion regulation, including distraction techniques, social connection with others, and engaging the mind:

*For me, that [referring to an online creative writing course] has really helped me in my mental health journey through the pandemic, and obviously, through my personal journey from my lived experience of having delayed surgery because of the pandemic and then having three surgeries within two months*… *I suffer very badly with anxiety, I have complex post-traumatic stress disorder, and I also experience psychosis. So having something where you are actively involved, not focusing on your own internal experiences, and that mental dialogue of challenging yourself all the time about what actually is happening around you. Focusing on a group setting where, because we haven’t been socialising, just with people on the screen and in the environment, where you are really focusing to share on their words, on the words of the lecturer who is facilitating session, and on the actual creative process yourself, it was obviously a really, very good distraction, a way from out of myself* (SU1).

*By being silly and using their imagination, people laugh and then their confidence grows… There’s the immediate impact of laughter and achieving things that they probably didn’t think they could… I think what arts can do for mental health is phenomenal… People will talk about how improv[isation] saved their life, literally… The more people that engage with art for mental health provision, the better because we in the arts know how powerful arts provision/arts participation is, whether that’s drama, craft making, singing, we know how powerful it is* (CP3).

Participating in the arts also supports the wellbeing of informal carers. One service user reported engaging in creative and cultural courses as a means to break up the stresses and strains associated with being a carer:

*As a carer you just need a break from constant worry, organising things, putting somebody in front of yourself all the time, somebody else’s needs. I thought this is something for me to sit down, something to make and lose myself in because I haven’t done crafts since I was at school* (SU5).

*This is our little sanctuary… We like to close the door on the outside world… This is my little oasis of getting together and it’s a lovely feeling* (CP1).

As attendance was becoming part of people’s ongoing social life, such courses fostered feelings of belonging:

*We work together to explore different ways of making music together. But it’s more perhaps about the being in a group that comes from that, rather than the musical result* (CP6).

*With the storytelling course they were all given an opportunity to celebrate their individuality, but at the same time to feel part of a group* (CP2).

The acquisition of new skills and sense of accomplishment through skill building was also highlighted. Users of the Life Rooms found value in learning new capabilities, such as poetry writing, whilst also building confidence in taking risks and engaging in novel activities, as well as enhancing self-esteem through the experience of personal accomplishment:

*My knowledge about poetry and creative writing was zilch, so I was starting from the very beginning. So, it was really a lovely process for me… I think it was therapeutic, because it was an education. We were learning* (SU3).

*When you learn something, there’s a sense of achievement… Today there were three dances that they have never done before… When you learn about it step by step and then it culminates into something there’s this sense of achievement, feeling good about themselves* (CP4).

*I found it really beneficial for my confidence because [although] I write poetry sometimes, I’ve never actually spoken it in front of someone, so it was the first time I have ever done that yesterday. It was a really good experience. I think going forward that will help me not only to attend courses, but also, it’s almost like a transferable confidence skill* (SU4).

Further supporting this, courses facilitated by some of Mersey Care’s cultural and creative partners, such as Liverpool Philharmonic, culminate with a live performance at a different, prestigious venue. Working towards a live performance gave service users a sense of satisfaction and pride:

*At the end of the course, we put together a little show, and that’s on at the Liverpool Phil[harmonic] itself with players from the Phil[harmonic], so the participants get an opportunity to perform with a professional backing group* (CP5).

*It’s an outing where they can invite family and friends, and it just brings people together in a prestigious venue in the city centre… It’s been just incredible to see the levels of confidence building up towards that event… to be in front of an audience and to present something that is really personal and that is really meaningful to them… Once the final event is there, and you throw yourself on that stage, it’s a massive sense of achievement, which obviously goes with your self-esteem* (CP2).

### Role of creativity and the arts beyond the pandemic: ‘It’s all about connecting people’

Creative practitioners emphasised the impact of the COVID-19 crisis on people’s mental health and wellbeing (‘*The impact that the lockdown had on people, it’s deep. It’s really deep’* (CP4)). As the ways in which the COVID-19 pandemic has affected individuals will be felt in the months and years to come, mental health demand could continue to grow, even as the pandemic has abated:

*Not in our lived experience or lived memory have we ever experienced this. So, I think the trauma from that is huge… Coming out of a pandemic, going forward to recover from a pandemic is going to take even more intervention, whether that’s mental health, stress reduction or art for confidence or drama, whatever. It’s like the demand is never going to go away, and it’s probably got worse* (CP3).

Creative practitioners emphasised that the arts could have an important role in meeting mental health needs and helping people to recover from the pandemic as the social ramifications continue to be felt:

*Our whole way of living and relating to people in a social setting has completely changed. One of the impacts is social interaction. And I think that is where drama and arts-based activities can really help. It’s like, how do we look at people in the eyes again? How do we listen well? How do we communicate to each other? Essentially drama is a communication tool. So of course, going forward, participation in the arts can help with social skills and confidence and communication skills, and all of that. But it can also help with self-expression… There are ways of self-expressing through drama, through art, that is going to be essential for people’s recovery. But on top of that, we need to learn how to connect and relate and communicate to each other again* (CP3).

*It’s really just a matter of utilising it [referring to the arts] to tackle different things. For example, if somebody’s got long COVID, who is slowly reintroducing into being in spaces with people, it’s a good thing to do. It’s a good activity [referring to creative writing]. It’s a good way of making someone feel positive about themselves, giving them an opportunity to think about what’s happened to them and write about that and rationalise that or like find a way through it* (CP8).

Thus, in the wake of the crisis caused by the pandemic, the role of arts and culture in providing stigma-free environments to reconnect the vulnerable and isolated, and promote the sense of belonging described above, is more critical than ever:

*Feeling part of something is so important in terms of this recovery, the recovery period where people felt so isolated at home, that they can feel actually part of a team / part of a group, and they belong somewhere* (CP5).

*It’s all about connecting people, and it’s a lovely way into a relationship with somebody. It’s not necessarily about arts and culture, it’s more about just relationships*… *I do feel that having been isolated in every way during a pandemic it is important more than ever to connect more and create these bonds and relationships with people so we can get that community again* (CP6).

The importance of reconnecting with others following extended periods of isolation was also recognised and appreciated by users of the Life Rooms:

*People are struggling but even more so now. For people like me, any opportunity to get out and meet people, learn new skills, get our confidence, and give us our headspace back because the mental pressure for the last two years for me has been incredible… Loneliness is bad at any time, but I think a lot of people have been extra lonely during COVID. They haven’t been allowed to meet people or travel or even see family. I think you’ve just got to reach out for your mental health* (SU5).

## Challenges with the Life Rooms model in the new normal

### Insufficient resources

Some creative practitioners have encountered challenges whilst delivering their courses within the Life Rooms. In particular, issues with staffing capacity were highlighted, which negatively impacted the delivery of in-person sessions:

*I email the resource over to the life learnings [team]and one week they were very, very short staffed and whoever was on couldn’t find the email with the story and the poem, and I hadn’t brought anything with me* (CP1).

When services moved entirely online during the pandemic, Life Rooms learning facilitators – for the first time - attended all sessions delivered by creative practitioners as an extra layer of safeguarding. The presence of a Life Rooms learning facilitator was an innovation appreciated by creative practitioners:

*When we were on Zoom, there was a real system where there was always a facilitator assigned to the session, who would attend the whole session and who would be aware of what was happening and therefore could sustain in a way the activity by making sure emails were forwarded [to participants]… Unless I have a Life Rooms assistant when I’m delivering my courses back in real life, it’s going to be difficult to maintain these ways of communicating and sustaining the activities throughout the week… [with] gaps in the communication chain* (CP2).

Staff buy-in is important as Life Rooms staff members are critical to encouraging service users to take part. Thus, it was suggested that the Life Rooms learning facilitators could be given permission to participate in creative and cultural offerings provided by partner organisations in-person as, according to creative practitioners, such support ‘*would make a big difference*’ (CP2):

*There are some [learning] facilitators at the Life Rooms who have experienced drama, because they were involved in the online… For me, I think that’s really important (i) it shows participants a model, it models the behaviour, it models engagement, and (ii) the facilitators then get lived experience of sessions, that they can then communicate to other people and say, ‘Oh, look, I’ve done this session, and it’s really fun, and it’s really doable. You don’t have to be a performer’. It’s all part of getting participants engaged and to participate in the arts* (CP3).

*The more we work closely with each other the better. I’m a big advocate of running sessions where everybody’s on an email, coproduce, collaborate* (CP2).

As Life Rooms learning facilitators currently are unable to support creative courses delivered in-person, practitioners expressed concerns around managing the complexity and risk that can arise. They stressed the value of the presence of a Life Rooms learning facilitator supporting each session and providing assistance if a participant becomes unwell or distressed:

*There were other people who were in a very vulnerable state, and I just thought, I’m not equipped to deal with it* (CP6).

It is important to consider the wellbeing and safety of practitioners, especially as they are working with people who may have complex mental health needs. In addition to being paired with a Life Rooms learning facilitator, prior training needs were also identified to ensure that practitioners are fully equipped to address any issues that arise whilst delivering courses at the Life Rooms. In particular, it was felt that practitioners should receive training in mental health first aid, as service users were not permitted to meet – and effectively continue the arts activity on their own – without either a creative practitioner or Life Rooms facilitator present:

*Prior to COVID, I was delivering sessions in Bootle and the group was a lovely group, and they really wanted to carry on doing sessions after the course had finished so I said well, I’ll speak to the Life Rooms staff to see if this room is available because maybe you could keep meeting at the same time. When I suggested that to the Life Rooms staff, they said no, the group can’t meet without [either a Life Rooms facilitator or creative practitioner] present. So, I realised that actually my being there was providing something other than musical, but it wasn’t made clear to me what that was*… *I’ve had no mental health training. Independent of this [referring to Life Rooms provision], I’m actually doing a psychotherapy Master’s, so I have some kind of education, but that’s not equipping me to deal with people in crisis* (CP6).

Last, concerns were also shared around funding, particularly the lack of long-term security. Freelance practitioners require funding to deliver creative courses; however, one participant reported that provision had been cut back in some circumstances:

*Sometime in the Autumn 2021, the Life Rooms came back into their in-person provision. I went to every Life Rooms campus so Walton, Southport, Bootle. I rotated once a week through the campuses. Then that ended at the end of December, I think they ran out of funding, or they weren’t sure what the provision was going to look like in January 2022 so there was a bit of a hiatus. And then they brought me back again just for Walton this time, once a week* (CP3).

### Marketing and promotion

Following the reopening of the Life Rooms, as identified above, only a limited number of people have been attending the cultural and creative courses, both online and in-person. Participants suggested that this could be due to a lack of effective marketing or promotion of these courses:

*I mean numbers [attending in-person sessions] are still relatively small… I’m not privy to how they [the Life Rooms] recruit and promote their services. But I just experienced low numbers… If I was in any way a decision maker at the Life Rooms or Mersey Care, I would have a designated, dedicated publicity machine because what they do is so important… The way people know about stuff is you’ve got to flood social media and flood the website; you’ve got to promote your services* (CP3).

In fact, some users of the Life Rooms had not been aware that the Life Rooms service was restructured during the pandemic to provide a full suite of online courses:

*Of the attendees that were there, there was only one of them who was a regular of the online sessions. I think she was the only person there who knew the online sessions existed at all. So, most of the group were really surprised that these things had been running all through the pandemic* (CP8).

This highlights the need for the Life Rooms to explore ways to increase awareness, course attendance, and footfall at the centres. Creative practitioners suggested advertising more widely to promote the service throughout the city region, including via social media channels to raise the profile of the service:

*I think the main challenge is drumming up the support so getting the numbers in there… I think it’s recruitment into the course* (CP5).

*If you’re like a smaller business or a more responsive business, you’d have your social media marketing team come in and do a little video, do a Vox Pop. I know there’s probably confidentiality issues, so you will have to get consent from participants… I think you need to pay people to promote the services, especially now going forward, people are struggling with mental health*… *You’re never going to reach people unless you actually really advertise and evaluate. Capture evaluation, use it to promote. I think everyone should know about the Life Rooms and the other organisations that are trying to alleviate mental health stresses using art* (CP3).

### Inadequate feedback mechanisms

Some creative practitioners expressed concerns with the feedback mechanisms in place at the Life Rooms. At present, the Life Rooms use a learning evaluation form to collect feedback from users:

*[The form asks] which course have you attended today? Name? Date? But I don’t know what they do if they just give the form or if they sit down with them and discuss the impact that it had in their life a little bit more, not through form but face-to-face. Sometimes when you do things on a form, people take the form, and they don’t do anything, and they just leave it* (CP4).

In order to understand the sustainable impact of the creative offer, it would be beneficial for the Life Rooms to routinely collect detailed qualitative data from users to better understand the ‘qualitative worth’ (CP3) and effectiveness of each creative course. Creative practitioners suggested that this data could be collected during a reflective group session following the conclusion of each course:

*What people say in session at the end when we do a checkout is absolutely beautiful. When I say ‘how was that for you? And what did you enjoy? And how do you feel right now?’ It’s beautiful what people say, but there’s no way of capturing that. Again, where’s the camera capturing that on video and sticking it out on the website?* (CP3).

*I always thought of asking [Life Rooms staff], when people participate in the class, do you get a wrap up of getting them together and asking what did you enjoy? And what they felt?* (CP4).

It was recommended that the Life Rooms should collect such individual experience data during a reflective practice session using a variety of different mediums, such as written, audio, and video:

*It’s about being inventive and creative about how you get that feedback. It could be an audio, every phone has got a voice recorder on, do a little audio, and then get the marketing department to edit an audio of like feedback, and then whack it on the website and on the Facebook page… If I’m suffering from lack of wellbeing or mental health issues, and I come across the Life Rooms on the Facebook, I want to hear what other participants are saying, because that will give me courage to engage. If I hear a video or podcast, anything that is about, ‘oh my god, that was brilliant, I had such a laugh’, or ‘oh my god, feel like I could do anything now’, or ‘it was so nice to connect with people’, if I’m hearing that, then I’m going to go, I think that sounds ace, I want to do this… because these other people are loving it and I want that*… *If it reaches just one more person to enable them to step through the door of the Life Rooms and engage, that’s a win for me. That’s a total win* (CP3).

## Discussion

The present study explored the impact, from the service user and practitioner perspective, of the return to in-person provision at the Life Rooms, as well as the relative merits of online and in-person provision. Our key findings fall into three main categories: provision in the ‘post pandemic’ world, impacts, and challenges with the Life Rooms model in the wake of COVID-19.

Our findings illustrate the critical role of creative and cultural courses in providing stigma-free environments to reconnect the isolated. In particular, users of the Life Rooms appreciated the surrounding social experiences associated with in-person provision, such as opportunities to share experiences and interact with similar others. Yet, as re-engagement remained slow, there is a need to be responsive to hesitation around the return to in-person provision. In line with previous research (e.g., [19]), online engagement was used as a tool to support mental health and wellbeing during the pandemic, and this form of provision remains vital for those who are unable to leave their home due to health or mobility issues. Thus, our findings show a demand for maintaining online provision that enables accessibility together with in-person provision that boosts community connectedness.

Previous research has shown that engaging in participatory arts activities can promote mental health and wellbeing (e.g., 4), and our findings support this by providing evidence of the role of community arts provision in enhancing wellbeing. As engagement with the arts can have preventative benefits, including reducing the impact of trauma [5], creative practitioners highlighted the importance of engaging in in-person participatory arts activities following the crisis as a safe means of reconnecting with others and a means of processing negative experiences or emotions aroused by the pandemic.

While the Life Rooms is an established and successful model of integrating arts and culture within NHS provision [16], our findings suggest that arts-in-health partnership working under the Life Rooms model faces challenges in the context of the new normal. Our participants highlighted insufficient resources, issues with marketing and promotion of creative and cultural courses, and the need for improved feedback mechanisms. While these issues, especially those around staffing and marketing, are closely linked to the post-pandemic climate, particularly the increased pressure on healthcare services in the North since the onset of the COVID-19 pandemic [20], a number of implications arise from the study. First, our findings highlight the importance of teamwork as having a Life Rooms learning facilitator present during online sessions was identified as a factor facilitating effective delivery. It is also crucial to consider the wellbeing and safety of practitioners, as they are working with people who may require emotional support [21]. Creative practitioners could therefore be paired with a Life Rooms learning facilitator when delivering courses at the Life Rooms. This is particularly recommended as creative practitioners found this practice to be a valuable addition to usual support when facilitating sessions via online means. Practitioner safety and wellbeing could also be better secured by practitioners having access to training in mental health first aid. Second, again building on a need/priority identified in previous research [22], in order to understand the sustainable impact of the creative offer, the Life Rooms could routinely collect rich qualitative data (written, audio, and/or video feedback) from those who participate in creative courses to capture the impact of regular participation on wellbeing, especially as individual experience data could be used to aid marketing and promotion of courses. Relatedly, our findings highlight the importance of listening to service users and regularly seeking their engagement preferences, especially as there are mixed attitudes towards in-person engagement [23]. In light of these concerns, the Life Rooms could consult service users via an anonymous survey to identify and accommodate their preferences. There are implications for the delivery of future provision, with potential for online offerings to continue alongside in-person courses. Future research should therefore compare the efficacy of online and in-person creative and cultural courses, especially as creative practitioners highlighted that online engagement may not provide as strong wellbeing benefits as in-person engagement [24].

These findings should be considered in light of a number of limitations. As only five users of the Life Rooms participated in the study, the views from these individuals may not be representative of the experiences of all individuals who access Life Rooms provision. Nevertheless, mixed views on provision format were elicited. Similarly, as only eight creative practitioners participated in the study, our findings are limited to those individuals and may not be representative of the views held by all practitioners who deliver courses at the Life Rooms. Future research should seek to incorporate the views of NHS representatives, such as Life Rooms learning facilitators. All interviews were conducted between March and April 2022, and therefore do not capture experiences outside of this timeframe. It is likely engagement preferences might fluctuate depending on season and the number of COVID-19 cases.

## Conclusions

To conclude, our study shows a demand for maintaining online provision that enables accessibility together with in-person provision that boosts community connectedness. Although there has been a decline in the number of people accessing online provision, this format remains vital for those who are vulnerable, isolated, or otherwise unable to access in-person activities. As mental health demand could continue to grow in coming years as the pandemic evolves, arts and cultural engagement could play an important role in meeting mental health needs.

### Electronic supplementary material

Below is the link to the electronic supplementary material.

Supplementary Material 1

Supplementary Material 2

## Data Availability

Qualitative data extracts are presented in the article to support the findings. The data generated and analysed during the current study are not publicly available as the data collected is sensitive and could compromise the confidentiality and anonymity of the participants but are available (limited) from the corresponding author on reasonable request.

## Footnotes

1 This study is an extension of existing research activity undertaken as part of an AHRC-funded study, Covid-19 CARE (Culture and the Arts, from Restriction to Enhancement: Protecting Mental Health in the Liverpool City Region). Working with 15 cultural partners and 3 local health bodies, the AHRC-funded study produced findings on the impact of alternative/online provision of arts and culture in the Liverpool City Region (LCR) on the region’s mental health as a result of Covid-19 restrictions. The data collected for our AHRC-funded study covered two time periods: (i) full lockdown (Winter/Spring 2020–2021) (ii) immediate post-lockdown (the transitional stage of restriction-easing, Summer 2021). The study also focused principally on arts-cultural providers, and their regular audiences/visitors, in relation to their experience of online/hybrid delivery of arts and culture. The present evaluation adds value to our previous findings by concentrating on the impact of in-person arts/cultural activity provided by five of our arts partners in collaboration with a mental health care provider (our partner, Mersey Care NHS Trust), as part of its pioneering Life Rooms offer.

2 The UK Government introduced the national shielding programme at the beginning of the COVID-19 pandemic, with individuals identified as clinically extremely vulnerable offered advice and support to stay at home and avoid all non-essential contact.
