# Supplementary material for: Re-engaging with arts and cultural activities at the Life Rooms: ‘It’s given me spring’
Source: BMC Complement Med Ther. 2024 Jun 15;24:235. doi: 10.1186/s12906-024-04539-6 (PMC11180401; doi:10.1186/s12906-024-04539-6)
Supplement: Supplementary file 1 — Supplementary Material 1 [file 12906_2024_4539_MOESM1_ESM.docx]

**Supplementary file 1**

**Example interview questions (beneficiaries)**

**Return to in-person provision**

Tell me about any activities/courses/groups that you have attended since the Life Rooms reopened. What was that like for you as a participant?

Why did you decide to attend this course or group (or these courses/groups)? / What motivated you to participate?

Which activities have you been most pleased to return to?

What do you enjoy about engaging in this activity/course? What benefits would you say you derive from it? / What do you like about attending these activities in-person?

Has participating again in in-person activities/courses/groups had an impact on your wellbeing? / What impact has the return to in-person provision had on your wellbeing?

How does current in-person provision compare to in-person provision before the global pandemic? Are there any differences (e.g., social distancing measures)?

Is there anything you would like to change? Any improvements or suggestions?

**Online provision**

Tell me about any online activities/courses/groups that you have attended at the Life Rooms (since the launch of the Life Rooms online in October 2020). What was that like for you as a participant?

Has participating in online activities/courses/groups had an impact on your wellbeing?

What do you like about online activities/courses/groups? / What works well online?

What do you not like about online activities/courses/groups? / What doesn’t work well online?

How does face-to-face provision compare to online provision? Which do you prefer and why?

Now the Life Rooms has reopened, will you continue attending online courses/arts activities online?

Do you have any additional comments?
