# Supplementary material for: Re-engaging with arts and cultural activities at the Life Rooms: ‘It’s given me spring’
Source: BMC Complement Med Ther. 2024 Jun 15;24:235. doi: 10.1186/s12906-024-04539-6 (PMC11180401; doi:10.1186/s12906-024-04539-6)
Supplement: Supplementary file 2 — Supplementary Material 2 [file 12906_2024_4539_MOESM2_ESM.docx]

**Supplementary file 2**

**Example interview questions (practitioners)**

**Current provision**

To what extent have activities returned to pre-COVID provision?

Can you tell me a little bit more about current (in-person) provision?

- How does current provision compare to pre-lockdown provision? Are there any differences?
- How does current (in-person) provision compare to online provision via Zoom (a) during COVID-19 lockdown (b) at the current time? [Prompt: What works better online? What works better in-person?]

To what degree have pre-lockdown audiences/service users/beneficiaries/members renewed their participation in your organizations’ activities (in-person)?

- How do participants feel about re-integrating in-person/engaging with arts activities in-person?
- [If applicable] Why have some participants not renewed participation (in-person)?
- How do you feel about in-person provision at the moment?

What has been the impact of renewed access to the arts on participants’ mental health and wellbeing? / What impact does being back together in a shared physical space have on people’s mental health and wellbeing?

Can you reflect on any successes?

Can you reflect on any challenges?

Can you tell me about the hybrid model? How is this working? [if applicable]

- Are some participants opting to continue participating online rather than return to in-person provision?
- Why have you opted for hybrid delivery? / What are the advantages of implementing a hybrid model?

**Role of arts and culture beyond the ending of restrictions in July 2021**

Do you think arts and culture will play a role in supporting people’s mental health and wellbeing beyond the ending of restrictions in July 2021? If so, what will you do to support people’s mental health and wellbeing during this period?

Do you have any concerns moving forward?

Do you have any additional comments? Is there anything else you would like to say?
